# Use of biosurfactants, microorganism-destructors, and plants for eco-friendly bioremediation technologies on oil-contaminated soils

**DOI:** 10.5114/bta/209980

**Published:** 2025-12-08

**Authors:** Andriy Banya, Oleksandr Karpenko, Tetyana Pokynbroda, Olena Karpenko, Vira Lubenets

**Affiliations:** 1 Department of Physical Chemistry of Fossil Fuels of the Institute of Physical-Organic Chemistry and Coal Chemistry named after L. M. Lytvynenko of the National Academy of Sciences of Ukraine, Lviv, Ukraine; 2 Department of Technology of Biologically Active Substances, Pharmacy and Biotechnology, Lviv Polytechnic National University, Lviv, Ukraine

**Keywords:** oil-contaminated soil, bioremediation, biosurfactants, microbial preparation, plants

## Abstract

**Background:**

Soil contamination by oil products is a significant problem that affects the environment, agriculture, economy, and human health, and requires effective solutions. The study aimed to develop effective methods of bioremediation of oil-contaminated soils using microbial preparation D (a mixture of *Rhodococcus* sp. and *Gordonia* sp. – a consortium of autochthonous hydrocarbon-degrading micro-organisms), a rhamnolipid biocomplex (RBC), the oxidant calcium peroxide (CaO_2_), and plant remediants.

**Materials and methods:**

Bioremediation processes were carried out on oil-contaminated clay soil (initial contamination – 9.5%) over 1.5 years. First, the soil was treated with microbial preparation D and CaO_2_. After 14 days, field peas or sorghum were sown, with seeds treated using an RBC solution. Hydrogen peroxide content and lipid peroxidation index in plants, as well as soil dehydrogenase activity, were determined by spectrophotometry. Additionally, soil phytotoxicity was assessed using test plants, and the residual content of oil products was quantified.

**Results:**

The best effect was achieved with the combined use of microbial preparation D, RBC, and CaO_2_: the degree of oil contamination in the soil decreased to 1.3%; with microbial preparation D, plants, and RBC, contamination decreased to 1.4–1.6% (compared to the initial 9.5%). The maximum value of dehydrogenase activity was recorded when sorghum, microbial preparation D, and RBC were applied, 2.7 times higher than in the control. After bioremediation, the phytotoxicity of oil-contaminated soils (in test plants) decreased on average by 3.7 times compared to the control.

**Conclusion:**

The effectiveness of the integrated use of hydrocarbon-degrading microorganisms, field peas, sorghum, RBC, and CaO_2_ in bioremediation of oil-contaminated soils was established.

## Introduction

Among the promising and ecologically acceptable methods of environmental restoration, priority is given to biological approaches (bioremediation, phytoremediation), i.e., the purification of soils and water using specific natural microorganisms and plants (Koshlaf et al. 2017; Rigoletto et al. 2020; Mishra et al. 2021). In biotechnology development, an important task is the creation of active microbial and plant agents, with preference given to consortia based on autochthonous microbiota isolated from contaminated sites and tolerant plants. Currently, bioremediation methods are widely used in global practice for *in situ* remediation of soils contaminated with petroleum products (Koul et al. 2018; Villalba Primitz et al. 2021).

Bioremediation is considered the most cost-effective technology for restoring technologically disturbed soils (Landa-Acuña et al. 2020), with costs ranging from $5 to $300 per cubic meter, depending on the method applied. In comparison, physico-thermal treatment and incineration cost about US $600 and US $2,000 per cubic meter, respectively, which greatly exceeds the cost of bioremediation (Bianco et al. 2023). The primary cost component of bioremediation depends on the type and level of pollution, as well as transportation and storage of bottom sediments for *ex situ* treatment.

However, even with active plants and microorganisms, bioremediation is often limited by the hydrophobicity and toxicity of pollutants and their low bioavailability due to strong sorption on soil particles (Souza et al. 2014; Jimoh et al. 2019; Gaur et al. 2021). In this regard, a pressing task is to develop complex remediation approaches, particularly through the use of effective stimulants. Such stimulants may include surface-active substances (surfactants), with the most promising being those of natural origin (biosurfactants) (Chaprăo et al. 2015). Comparable in effectiveness to synthetic surfactants, biosurfactants are, at the same time, environmentally friendly.

Due to their physicochemical properties (desorption of hydrophobic substances from soil, solubilization, and reduction of surface and interfacial tension of solutions), as well as their biological activity, biosurfactants can significantly enhance the efficiency of contaminant degradation and removal by microorganisms and plants (Galabova et al. 2014; Liao et al. 2016). Biosurfactants are widely studied in research on the bioremediation of soils contaminated with persistent pollutants such as hydrocarbons and heavy metals (Eras-Muńoz et al. 2022).

One of the best-known biosurfactants is rhamnolipids–glycolipids composed of one or two rhamnose units acetylated with up to three long-chain hydroxy fatty acids (Esposito et al. 2023). Most rhamnolipid biosurfactants are produced by bacteria of the genus *Pseudomonas* (Kashif et al. 2022). Their properties enable the solubilization of hydrophobic compounds in the aqueous phase, the formation of emulsions, and the modification of cell surfaces (Varjani and Upasani 2017). The presence of rhamnolipids improves the contact between microbial cells and hydrophobic organic pollutants, which in turn enhances the metabolic activity of hydrocarbon-degrading microorganisms and increases remediation efficiency (Khoshkholgh Sima et al. 2019). There are several possible mechanisms for organic pollutants biodegradation with rhamnolipids (Gaur et al. 2022). The first one is the solubilizing biosurfactant effect, which promotes the destruction of hydrophobic pollutants, increasing their bioavailability (Markande et al. 2021). The second is the promotion of microorganisms’ direct attachment to organic pollutants via modulating cellular hydrophobicity (Bao et al. 2022). Also, rhamnolipids affect growth and increase plant immunity (Crouzet et al. 2020). Such advantages of rhamnolipids can be used to improve soil remediation of various oil production objects.

Also noteworthy are chemical oxidants, in particular calcium peroxide, which can contribute to the primary oxidation of contaminants and simultaneously activate bioremediation by enhancing aeration (in soil or water), essential for hydrocarbon-degrading microorganisms. In addition, CaO_2_ absorbs carbon dioxide released during the oxidation of petroleum products, forming calcium carbonate, which helps improve the chemical composition of soil (Pagliarani et al. 2012).

In our study, bioremediation was applied to clean soils from real sites at the Oil and Gas Producing Department “Dolynanaftogaz” (Dolyna, Ivano-Frankivsk region, Ukraine). The experiment lasted 1.5 years, with an initial petroleum contamination level of 9.5%. The main advantages of bioremediation technologies are environmental safety, ease of application, and economic accessibility. *In situ* bioremediation is most effective at pollution concentrations up to 10%. For optimal results, it is considered necessary to assess the contamination status of a specific site and apply a combination of bioagents and activators. Therefore, the approach presented in this article is economically feasible for use on territories of real sites contaminated with petroleum products at concentrations up to 10%.

The aim of the study was to develop effective bioremediation strategies for the recovery of oil-contaminated soils using biological agents (microorganisms and plants) and activators (biogenic surfactants, oxidants) with different mechanisms of action, and to test their efficiency on soils from oil production sites.

## Materials and methods

To study the effectiveness of bioremediation, oil-contaminated clay soil from the area of the Oil and Gas Producing Department “Dolynanaftogaz” (Dolyna, Ivano-Frankivsk region) was used. The soil composition was as follows: clay – 56%, sand – 30%, silt – 10%, other – 4%; pH – 6–6.5, and oil content – 9.5%. The consortium of autochthonous hydrocarbon-degrading microorganisms (microbial preparation D) was used as a remediation agent, while field pea (*Pisum arvense* L.) and sorghum – sudan grass (*Sorghum bicolor* subsp. *drummondii*) were applied as remediation plants. As activators, the rhamnolipid biocomplex (RBC), a microbial synthesis product of the *Pseudomonas* sp. PS-17 strain (Semeniuk et al. 2020), and the chemical oxidant calcium peroxide (CaO_2_) (PIW “Impuls,” Poland) were used.

### Microbial preparation

Hydrocarbon-degrading microorganisms were isolated from soils with long-term oil contamination (Oil and Gas Producing Department “Dolynanaftogaz”) using the accumulation culture method (Segi 1983). The isolates were sequentially seeded on Shishkina-Trotsenko medium with crude oil, diesel fraction, or vaseline oil as carbon sources. Stable consortia of hydrocarbon-degrading microorganisms were obtained and further separated into strains. Their generic origins were determined through morphological and cytological studies. Primary identification was carried out by seeding onto selective agarized nutrient media. The resulting preparation D consists of a mixture of *Rhodococcus* sp. and *Gordonia* sp. – a consortium of autochthonous hydrocarbon-degrading microorganisms (1 : 1).

### Experimental design

A small-lot experiment was conducted on oil-contaminated soils of the Oil and Gas Producing Department “Dolynanaftogaz” over 1.5 years. The soil was pretreated with microbial preparation D at a ratio of 50 ml of microbial suspension (5 × 10^6^ CFU/ml) per 1 kg of soil. In one experimental variant, soil was treated with CaO_2_ at 3 g/kg, which was mixed with the entire soil volume. The prepared soil was left for 14 days, after which remediation plants – field pea or sorghum – were sown. Pre-sowing treatment of plant seeds was performed with the biosurfactant RBC (0.01 g/l) for 3 h, with water used as a control.

### Plant physiological and biochemical parameters

The hydrogen peroxide content was measured by a spectrophotometric method in plant homogenates after centrifugation (Chen et al. 1999). One milliliter of supernatant was mixed with 3 ml of 0.1% Ti(SO_4_)_2_, and the color intensity was assessed at 410 nm using a Shimadzu UVmini-1240 spectrophotometer (Shimadzu Corp., Japan). The H_2_O_2_ content was expressed in mM/g of fresh weight.

Lipid peroxidation (LPO) in plant cells was evaluated by estimating the malondialdehyde (MDA) content based on its interaction with 2-thiobarbituric acid. This reaction produced a colored compound with an absorption maximum at 532 nm, which was measured spectrophotometrically (Bagnyukova et al. 2007).

### Soil analysis

#### Analysis of residual oil-contaminated

Soil samples after the bioremediation process were extracted with tetrachloromethane (or toluene) in a Soxhlet apparatus. The extract was purified from polar compounds using a chromatographic column with aluminum oxide, and the solvent was evaporated under vacuum. The residual oil content was determined gravimetrically (Lurie 1973).

#### Soil dehydrogenase activity

Dehydrogenase activity was determined by the colorimetric method with 2,3,5-triphenyltetrazolium chloride (TTC) (Casida et al. 1964). Soil samples (6 g) were incubated with TTC for 24 h, then extracted with acetone. Absorbance of the extracts was measured at 485 nm using a Shimadzu UVmini-1240 spectrophotometer (Shimadzu Corp., Japan). Dehydrogenase activity was calculated from the calibration equation according to the amount of 1,3,5-triphenylformazan (TPF) formed (µg TPF per gram of soil in 24 h).

#### Soil microorganisms

The number of soil microorganisms was determined using the serial dilution method according to Pasteur (Segi 1983). One gram of soil was aseptically introduced into a flask containing 50 ml of sterile water and mixed to obtain a suspension. After sedimentation, 1 ml was aseptically transferred into a test tube with 9 ml of sterile water to obtain a 1 : 1000 dilution. From the first test tube, 1 ml of the mixture was aseptically transferred into the second one, from the second one into the third one, etc., obtaining successive dilutions. From each test tube, 1 ml of suspension was aseptically added to a Petri dish (with 20 ml of nutrient medium), incubated for 5 days at 30°C, followed by the calculation of CFU (colony-forming units) based on the colony count according to dilutions.

#### Soil phytotoxicity

The phytotoxicity of oil-contaminated soils was assessed using the Berestecky method with germination tests of radish (*Raphanus sativus* L.) and garden cress (*Lepidium sativum* L.) in Petri dishes (7 days, 23–25°C, in the dark). Substrate humidity was maintained at 70–80% of total moisture capacity, with garden soil used as a control. Seed germination capacity and seedling morphometric indices (length and mass of roots and shoots) were recorded, and the phytotoxic effect (PE, %) was calculated (Berestetskiy 1971).

### Statistical analysis

All experiments were performed in triplicate, and results are presented as mean values ± standard deviations (*n* = 3). Experimental data were processed using Microsoft Excel 2010. Differences between experimental groups were further analyzed with the Statistica software package, version 12.0 (StatSoft, Tulsa, OK, USA). Differences were considered statistically significant at *p* < 0.05 (Kucherenko et al. 2001).

## Results and discussion

**Figure 1:**
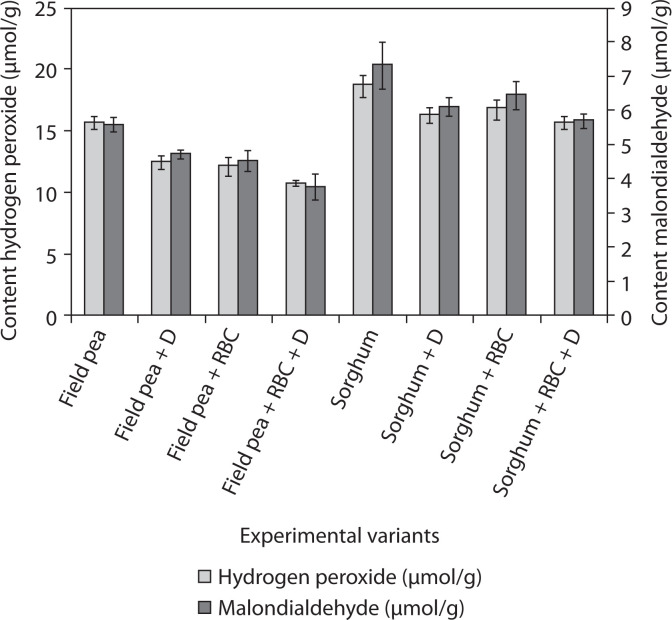
The content of hydrogen peroxide and malondialdehyde in field pea and sorghum after 3 months bioremediation of oil-contaminated soil from the area of the Oil and Gas Producing Department “Dolynanaftogaz”; RBC – rhamnolipid biocomplex (0.01 g/l); D – microbial preparation; initial content of oil-contaminated – 9.5%

Oil contamination of soil significantly affects plant growth, biochemical indicators, and adaptation to environmental conditions (Gospodarek et al. 2021). To counter the negative impact of pollution, plants can activate a complex of biochemical and physiological processes. These include removal, conjugation into intracellular compounds, compartmentalization of conjugates in cells, decomposition, transformation of pollutants into standard metabolites, or their mineralization (Kvesitadze 2013). For modern bioremediation technologies, the selection of effective remediation activators is an important task. To evaluate the intensity of redox processes that characterize the negative impact of environmental factors on plants, the content of hydrogen peroxide and malondialdehyde was determined (Figure 1).

The reduction of the studied parameters was observed in field pea and sorghum plants after the treatment of seeds with RBC solution: hydrogen peroxide content by 46% and 19%, respectively, malondialdehyde by 48% and 28%, if compared to the control (Figure 1).

Hydrogen peroxide acts as a second messenger in stress signaling and serves as an indicator of cell damage (Černý et al. 2018). Its accumulation can result from salt stress, chilling, mechanical damage, nutrient deficiency, pathogen infection, or environmental pollution (Khedia et al. 2019). Sanchez et al. (2012) demonstrated that rhamnolipid biosurfactants from *Pseudomonas aeruginosa* trigger an immune response in *Arabidopsis thaliana* by inducing the accumulation of signaling molecules and activating defense genes. According to Dupuy et al. (2016), elevated malondialdehyde levels disrupt the physiology of hydrocarbonstressed plants, ultimately inhibiting root growth. Similarly, El-Sheshtawy et al. (2022) studied the effect of biosurfactants from *Bacillus megaterium* used for presowing seed treatment on the growth and quality of *Lactuca sativa* under toxic exposure to heavy metals. Their findings showed that *B. megaterium* biosurfactants significantly improved morphological features, proline content, and antioxidant enzyme activity, while markedly reducing H_2_O_2_ levels and lipid peroxidation (El-Sheshtawy et al. 2022).

According to the obtained results, oxidative reactions in plants growing on contaminated soil were activated, as indicated by increased levels of MDA and H_2_O_2_, which may reflect a reduction in the overall impact of pollution. After presowing seed treatment with RBC solution, these parameters significantly decreased, suggesting improved adaptive capacity of plants to contaminants. These findings are consistent with our previous laboratory studies on the effects of biosurfactants on plant growth in model oil-contaminated soils (Banya et al. 2015; Karpenko et al. 2015). Moreover, the reduction in oxidative reactions was more pronounced in field pea than in sorghum plants (Figure 1).

To evaluate the effectiveness of the developed bioremediation approaches, the influence of biological factors and stimulants on the remediation of oil-contaminated soils (initial oil content 9.5% w/w) at the facilities of the Oil and Gas Producing Department “Dolynanaftogaz” was tested in a small-lot experiment. The main parameters used to assess remediation effectiveness were residual oil content in the soils, dehydrogenase activity, and the number of soil microorganisms. These indicators were measured three months after the first treatment of contaminated soil (Table 1).

**Table 1:** Results of complex bioremediation of oil-contaminated soil at the facility of the Oil and Gas producing department “Dolynanaftogaz”

Experimental variants	Oil-contaminated content in soil (% w)	Soil dehydrogenase activity (μ g TPF/g of soil)	Number of hydrocarbondegrading microorganisms (CFU/g of soil)	Number of eterotrophic microorganisms (CFU/g of soil)
Control	8.9	80.2 ± 2.9	3 × 10^4^	6 × 10^7^
Field pea	7.5	126.6 ± 4.7	2 × 10^5^	12 × 10^8^
Field pea + D	6.9	188.3 ± 5.1	2 × 10^6^	5 × 10^8^
Field pea + RBC	6.5	99.9 ± 2.3	8 × 10^5^	8 × 10^7^
Field pea + D + RBC	6.5	192.9 ± 3.7	5 × 10^6^	4 × 10^8^
Sorghum	8.1	87.8 ± 1.5	6 × 10^4^	4 × 10^6^
Sorghum + D	7.7	119.3 ± 2.5	9 × 10^5^	2 × 10^8^
Sorghum + RBC	7.9	127.4 ± 3.2	3 × 10^5^	8 × 10^7^
Sorghum + D + RBC	7.8	233.8 ± 6.8	2 × 10^6^	4 × 10^8^
D + RBC + CaO_2_	5.9	199.1 ± 3.7	7 × 10^6^	7 × 10^8^

RBC – rhamnolipid biocomplex (0.01 g/l), D – microbial preparation, initial content of oil-contaminated – 9.5%.

According to the data (Table 1), after 3 months of bioremediation, all experimental variants showed a reduction in oil content compared with the control. The greatest effect was achieved with the combined use of microbial preparation, biosurfactants, and CaO_2_, where the residual oil content decreased by 50.8% compared with the control. This effect of surfactants can be attributed to the solubilization of hydrophobic contaminants and their ability to increase microbial cell membrane permeability and enzyme activity (Eras-Muńoz et al. 2022).

Soil oil contamination is associated with water deficits in plants grown under such conditions (da Silva Correa et al. 2022). Changes in soil water–air properties lead to the formation of an impermeable oily film that surrounds seeds and prevents germination (Zió³kowska et al. 2010; da Silva Correa et al. 2022). The degradation of oil contaminants is further enhanced by CaO_2_, which, according to the literature, promotes partial oxidation of pollutants, improves soil aeration, and thereby stimulates microbial remediation (López et al. 2009; Karpenko et al. 2009).

In field experiments, Gargouri et al. (2013) and Bello-Akinosho et al. (2017) reported that consortia of different microorganisms exhibited significant hydrocarbon removal efficiency in contaminated soils. In our study, a significant improvement effect was also achieved through the gradual application of microbial preparation in combination with the sowing of field pea, whose seeds were pretreated with RBC solution.

Another important indicator characterizing the intensity of the remediation process and the “health of the soil” is soil dehydrogenase activity. In all experimental variants, dehydrogenase activity showed a significant increase: field pea + D + RBC – 2.2 times higher, sorghum + D + RBC – 2.7 times higher, and D + RBC + CaO_2_ – 2.3 times higher compared with the control. This reflects an increase in the functional activity of the soil biota, particularly hydrocarbon-degrading microorganisms.

**Figure 2:**
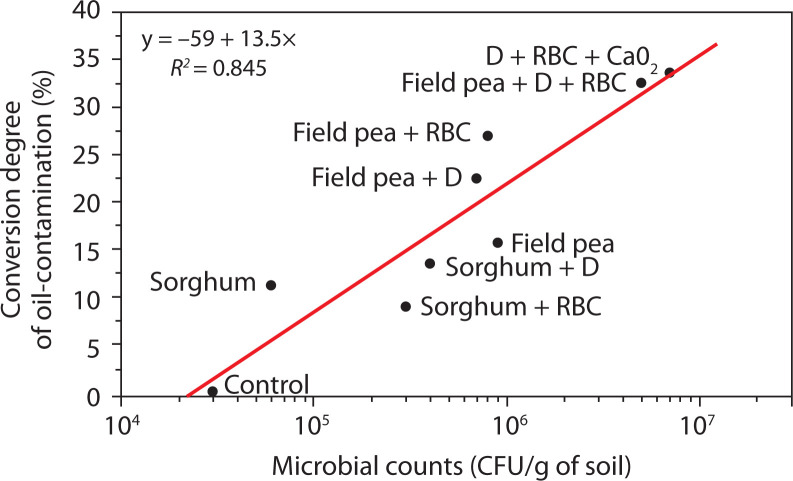
Relationship between oil-contaminated content and the number of soil microorganisms after 3 months bioremediation of oil-contaminated soil. RBC – rhamnolipid biocomplex (0.01 g/l), D – microbial preparation, initial content of oil-contaminated – 9.5%

Another key parameter of the remediation process is the total number of microorganisms, including hydrocarbon degraders (Table 1, Figure 2). The best results were obtained in the variants with microbial preparation combined with plants (5 × 10^6^ CFU/g) and with microorganisms + biosurfactants + CaO_2_ (7 × 10^6^ CFU/g) (Sihag et al. 2014). Literature indicates that for effective hydrocarbon biodegradation, the population of soil bacteria typically ranges from 10^4^ to 10^7^ CFU/g, while levels below 10^3^ CFU/g correspond to lower biodegradation potential. An increase in the hydrocarbon-degrading microbial population significantly enhances both the rate and efficiency of biodegradation (Roy et al. 2018; Varjani et al. 2019). Based on the obtained results, field pea proved to be the most tolerant and promising remediation plant and was therefore used in subsequent stages of soil purification.

**Figure 3:**
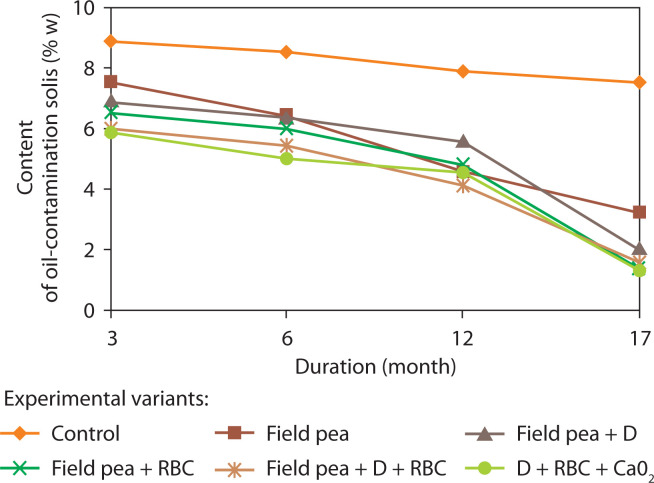
The dynamics of the oil-contaminated content in soil of Oil and Gas Producing Department “Dolynanaftogaz” in the complex bioremediation. RBC – rhamnolipid biocomplex (0.01 g/l), D – microbial preparation, initial content of oil-contaminated – 9.5%

A linear relationship was established between the change in oil pollutant content and the number of soil microorganisms (Figure 2) in the variants with microbial preparation, plants, and biosurfactant. This effect can be attributed to the stimulation of plant root system growth by rhamnolipid biosurfactants under oil-contaminated conditions (Banya et al. 2015; Karpenko et al. 2015). As the root system develops, it releases exudates (sugars, amino acids) into the soil, which are metabolized by soil microorganisms. This, in turn, influences both the abundance and taxonomic diversity of microorganisms in the rhizosphere (Correa-García et al. 2018; Vives-Peris et al. 2020). Such interactions may also indicate enhanced hydrocarbon degradation and improved soil microbiota activity, serving as markers of improved soil quality. Monitoring of oil-contaminated soils over 17 months of remediation showed that all applied combinations of biological agents and activators were effective (Figure 3).

At the first stage of soil remediation, the greatest reduction in oil product content was observed in the variant with microbial preparation and field pea (seeds treated with RBC solution). After 12 months of remediation, the best results were obtained with the combination of microorganisms, biosurfactant, and CaO_2_, where the hydrocarbon content decreased to 4.5% (Table 2). However, after 17 months of the experiment, the residual oil content also decreased significantly in other variants with plants, microbial preparation, and biosurfactants.

**Table 2:** Phytotoxicity of soil from the Oil and Gas producing Department “Dolynanaftogaz” after the complex bioremediation

Bioremediation variants					
Presowing treatment of seeds	Application to the soil	*Raphanus sativus*		*Lepidium sativum*	
Control	–	85	–	90	–
**Oil-contaminated soils**					
H_2_O	–	40	49	16.6	61.2
Field pea + H_2_O	–	70	24	80	43.5
Field pea + RBC	–	90	16.6	80	22.5
Field pea + H_2_O	D	70	34	83	19.3
Field pea + RBC	D	75	12.5	80	17.7
**Oil-contaminated soil without plants**					
–	D + RBC + CaO_2_	70	16.6	80	25

RBC – rhamnolipid biocomplex (0.01 g/l), D – microbial preparation, initial content of oil-contaminated – 9.5%.

After 17 months, the hydrocarbon content in soil decreased by 3–7 times compared with the initial level (Figure 3). The lowest residual oil content was recorded with the combined use of microorganisms, RBC, and CaO_2_, where contamination was reduced to 1.3%. A significant decrease was also achieved with microbial preparation, plants, and RBC, with oil content reduced to 1.4–1.6%, confirming the effectiveness of these components in soil remediation (Figure 3).

An important integrated ecological criterion for remediation is the reduction of soil toxicity, particularly phytotoxicity (Beshley et al. 2014; Lee et al. 2020). The toxicological assessment of oil-contaminated soils after complex remediation was conducted using radish (*Raphanus sativus* L.) and garden cress (*Lepidium sativum* L.) as test plants (Table 2).

It was established that the use of plants, biosurfactants, and microbial preparations reduced soil phytotoxicity. Radish seed germination increased 1.8-fold and garden cress seed germination 4.8-fold compared with the control (Table 2). Similar results were reported by Das et al. (2018) and Tang et al. (2011), who found that biosurfactants improve seed germination rates. The phytotoxic effect of the soil also decreased: for radish by an average of 3.9 times and for garden cress by 3.5 times compared with the control (Table 2).

Thus, the developed technology for complex remediation of oil-contaminated soils employs microorganisms (a consortium of autochthonous hydrocarbon-degrading strains) and remediation plants (field pea and sorghum – sudan grass) as the main biological agents. Biosurfactant (RBC) and oxidant (CaO_2_) serve as stimulants to enhance remediation efficiency. In our view, biosurfactants can influence all stages of the remediation process: they increase contaminant bioavailability for microorganisms and plants, facilitate their transport into cells, and stimulate plant growth. The application of biosurfactants also increases plant tolerance to pollutants, resulting in more effective remediation. Furthermore, this approach may be applied to greening settlements negatively affected by industrial emissions. The proposed biotechnology can contribute to ecosystem restoration and, consequently, improve public health.

## Conclusions

The combined use of microbial preparation D (a mixture of *Rhodococcus* sp. and *Gordonia* sp. – a consortium of autochthonous hydrocarbon-degrading microorganisms), remediation plants (field pea, sorghum – sudan grass), and activators – RBC and CaO_2_ – proved effective for the remediation of oil-contaminated soils. The best results were achieved with the combined application of microbial preparation D, RBC, and CaO_2_, as well as through stepwise soil treatment with microbial preparation followed by the sowing of plants (field pea, sorghum).

Soil dehydrogenase activity increased significantly: field pea + D + RBC by 2.2 times, sorghum + D + RBC by 2.7 times, and D + RBC + CaO_2_ by 2.3 times compared with the control, indicating enhanced functional bioactivity of the soil biota. The degree of initial soil contamination (9.5%) decreased in variant field pea + D + RBC to 1.3%, and with microbial preparation, plants, and biosurfactant, to 1.4–1.6%. Also, after bioremediation, soil phytotoxicity indicators decreased: with field pea, microbial preparation, and biosurfactant germination improved for garden cress by 4.8 times compared to the control. The phytotoxic effect on the soil also decreased: with radishes by an average of 3.9 times, garden cress by 3.5 times, compared to the control. The developed technology was tested on the territory of the Oil and Gas Producing Department “Dolynanaftogaz,” demonstrating the prospects of this integrated approach. Thus, the biotechnological potential of biosurfactants, microbial preparations, and plants for the remediation of technogenically contaminated soils has been confirmed. The proposed complex technology may be applied for the restoration of areas impacted by oil production, processing, and transport enterprises, and may also be valuable in emergencies (e.g., military operations, terrorist attacks, accidents).
